# Myeloid cell biology and inhibition of anti-tumor immune responses by MPDL3280A in urothelial bladder cancer

**DOI:** 10.1186/2051-1426-2-S3-P131

**Published:** 2014-11-06

**Authors:** Yuanyuan Xiao, Christina Rabe, Marcin Kowanetz, Thomas Powles, Nicholas J Vogelzang, Daniel P Petrylak, Yohann Loriot, Mitchell Denker, Rin Nakamura, Qun J Wu, Teiko Sumiyoshi, Zachary Boyd, Siew-leng M Teng, Xiaodong Shen, Gregg Fine, Daniel S Chen, Priti S Hegde

**Affiliations:** 1 Genentech, Inc., San Francisco, CA, USA; 2 Roche Diagnostics GmbH, Germany; 3 Barts Cancer Institute, Queen Mary University of London, London, UK; 4 University of Nevada School of Medicine and US Oncology/Comprehensive Cancer Centers of Nevada, NV, USA; 5 Yale Cancer Center, New Haven, CT, USA; 6 Gustave Roussy, Villejuif, France

## 

Treatment options for metastatic urothelial bladder cancer (UBC) are limited. Mutational complexity is known to be high in UBC and may correlate with increased immunogenicity. MPDL3280A, a human PD-L1 monoclonal antibody containing an engineered Fc-domain designed to promote a Th1-driven response, has demonstrated a RECIST response rate of 43% in diagnostically selected, pretreated patients with UBC. A total of 68 patients (67 with efficacy evaluable) were enrolled in the UBC cohort of the Phase I study; 45% were PD-L1 IHC diagnostic positive as defined by expression of PD-L1 on ≥ 5% of tumor-infiltrating immune cells. In the prescreened UBC population, the prevalence of PD-L1-positive patients was 27%.

Comprehensive gene expression analyses of UBC tumors were conducted to interrogate the tumor immune microenvironment in PD-L1-positive tumors and to identify potential mechanisms associated with response or resistance to MPDL3280A. In this study, PD-L1-positive tumors exhibited a high prevalence of gene expression markers associated with T-effector cells (Teff), including perforin, IFNγ, CD8A, granzyme B, granzyme A and EOMES. Additionally, a low baseline signature of genes associated with myeloid cell markers, including *IL1B *and *IL8*, appeared to be statistically significantly associated (*P*<0.01) with MPDL3280A response, suggesting a potential role for myeloid biology in resistance to MPDL3280A treatment in UBC.

Tumor burden markers, including CA-125, CA19-9 and human chorionic gonadotropin (HCG), have been associated with chemotherapy response markers in UBC. A marked decrease in these markers, including CEA, CA19-9, CA-125 and HCG, was observed with MPDL3280A response after 1 treatment cycle, potentially enabling an on-treatment monitoring alternative for response to therapy. Similarly, evaluation of cytokines on treatment identified markers, including IL-6 and IL-10, elevated as early as Cycle 2 only in patients without response to MPDL3280A. These circulating cytokines and tumor-associated gene signatures suggest potential mechanisms associated with resistance and response to MPDL3280A in UBC and provide a rationale for informed combination strategies to further improve treatment benefit in this indication.

